# Mental Health Outcomes Across the Lifespan in Individuals With Persistent Post-Concussion Symptoms: A Scoping Review

**DOI:** 10.3389/fneur.2022.850590

**Published:** 2022-04-11

**Authors:** Elena Sheldrake, Hiba Al-Hakeem, Brendan Lam, Benjamin I. Goldstein, Anne L. Wheeler, Matthew Burke, Benjamin T. Dunkley, Nick Reed, Shannon E. Scratch

**Affiliations:** ^1^Bloorview Research Institute, Holland Bloorview Kids Rehabilitation Hospital, Toronto, ON, Canada; ^2^Rehabilitation Sciences Institute, University of Toronto, Toronto, ON, Canada; ^3^Centre for Addiction and Mental Health, Toronto, ON, Canada; ^4^Department of Pharmacology and Toxicology, University of Toronto, Toronto, ON, Canada; ^5^Department of Psychiatry, University of Toronto, Toronto, ON, Canada; ^6^Neuroscience and Mental Health Program, Hospital for Sick Children, Toronto, ON, Canada; ^7^Department of Physiology, University of Toronto, Toronto, ON, Canada; ^8^Sunnybrook Research Institute, Sunnybrook Health Sciences Centre, Toronto, ON, Canada; ^9^Institute of Medical Sciences, University of Toronto, Toronto, ON, Canada; ^10^Department of Paediatrics, University of Toronto, Toronto, ON, Canada

**Keywords:** concussion, persistent, children, adults, mental health, prevalence

## Abstract

**Objective:**

Concussion is a common yet heterogenous injury. Approximately 15–30% of cases present with persistent post-concussion symptoms (PPCS), continuing 4 weeks or more post-injury in children, youth, and adolescents, and 3 months or more in adults. There are known bidirectional links between PPCS and mental health outcomes. The focus of this scoping review is to explore the literature on mental health outcomes in individuals experiencing PPCS. Research objectives were to explore: (1) the mental health outcomes of individuals with PPCS and types of assessments used to identify mental health outcomes this group, and (2) how mental health outcomes compare in terms of similarities and differences among pediatric and adult populations with PPCS.

**Method:**

Ovid MEDLINE; EMBASE; CINAHL, and PsycInfo databases were searched. After title and abstract screening of 11,920 studies, 481 articles were reviewed. Twenty-five papers met inclusion criteria. Results were organized by mental health outcomes of pediatric and adult populations, separately.

**Results:**

There was a significantly higher number of studies devoted to adult populations. Of the 25 studies, 19 (76%) focused on adults, while six (24%) focused on adolescents. In adult populations, studies focused on symptoms of: anxiety (*n* = 2), depression (*n* = 8), and anxiety and depression (*n* = 9). Two studies assessed other emotional outcomes (10.5%). Within pediatric populations, an equal number of studies explored symptoms of: anxiety (*n* = 2), depression (*n* = 2), and anxiety and depression (*n* = 2). No studies focused on other emotional outcomes. Studies ranged greatly in methods, design, and control group. Most studies reported higher psychiatric symptoms of anxiety and/or depression in those with PPCS compared to individuals with recovered concussion or healthy controls.

**Discussion:**

This review contributes to the understanding of mental health outcomes in those experiencing PPCS. Mental health and PPCS requires greater attention in pediatric populations, and consider strategies for those experiencing PPCS and mental health impacts. Future studies should consider including a wider range of emotional outcomes in their design, not limited to anxiety and depression. Study results may lead to improvements and research in the identification, assessment, and management of PPCS and mental health.

## Introduction

Mild traumatic brain injury (mTBI), or concussion, is an international public health concern (1–5). Concussion is an injury induced by biomechanical forces, such as a blow to the head or body causing the brain to move rapidly inside the skull (6). Clinical presentation of symptoms following concussion are variable, and can manifest as a combination of cognitive, physical, affective, or sleep-related outcomes (7–9). Although most individuals who sustain a concussion recover without complications, studies show that symptoms can persist beyond 3 months post-concussion for 15–25% of adults (10–12) and beyond 4 weeks post-injury in ~30% of children (13). Hence, after sustaining a concussion, a sub-set of individuals may experience persistent post-concussion symptoms (PPCS). Common PPCS symptoms include headaches, dizziness, sleep disturbance, and psychological symptoms such as irritability, anxiety, and depression (14).

One challenge within this field is that there are several definitions of PPCS that differ within the adult and pediatric literatures and limited understanding for exactly which individuals will fall into the PPCS sub-set (15, 16). One definition requires adults (18+ years) to have a history of concussion, display cognitive impairments, and showcase at least three of the following symptoms for a minimum 3-month duration: fatigue, headache, dizziness, sleep disruption, irritability, apathy, or changes in personality (17). The Berlin Consensus Statement on Concussion in Sport defined a prolonged time frame to be more than 2 weeks in adults and more than 4 weeks in children (6). The specific symptoms or symptom profiles are not explicitly outlined within this definition. An expert consensus-based definition for persistent post-concussion diagnosis in those 16 years or older states that concussion symptoms must be present every day for 3 months after the trauma, and have an impact on at least one sphere of a person's life (18). Despite these differing definitions, there seems to be consensus that PPCS may have serious consequences for psychosocial-behavioral development (19, 20) and quality of life (21).

Individuals who have experienced a concussion may be at risk for poor mental health outcomes (22–24). A population-based study by Yang et al. (25) found that adolescents who experience a concussion are at an increased risk for higher depressive symptoms and self-injurious behaviors. Additionally, delayed symptom resolution has been associated with increased anxiety compared to children with early symptom resolution (26). Amongst adults, a study by Whelan-Goodinson et al. (27) found that >65% of those with concussion experienced symptoms of depression and anxiety after injury and showed poor recovery rates. For those with PPCS, new diagnoses of psychiatric symptoms have been reported in 36% of adolescents (28). In addition, pre-injury psychological and psychiatric history have shown to be predictors of PPCS (12, 29). Therefore, a link between concussion and poor mental health outcomes such as depression and anxiety (30) in both pediatric and adult populations has been established in the literature. There are three possibilities that may explain this link. First, concussion directly results in mood dysregulation and symptoms that impact mental health. The *Concussion in Sport Group Consensus Statement* highlights the importance of post-concussion mental health as mood problems like depression can develop after injury (6). Another possibility is that reduced activities associated with the post-concussive state have psychosocial implications that could lead to poor mental health outcomes. A third possibility is the link between pre-injury family and personal psychiatric history that can increase susceptibility to future mental health conditions post-injury (31). PPCS can make daily functioning significantly harder, thereby lowering quality of life in social, academic, and professional sectors (21). In addition, symptom overlap may partially explain this link, as irritability, anxiety, and difficulty concentrating, are a few of the symptoms found in those with PPCS and other psychiatric disorders (32). Thus, it is important to explore the relationship between mental health outcomes and PPCS for the early identification of mental health difficulties in individuals with PPCS.

Recent reviews have focused on the association between post-injury mental health and prolonged recovery in athletes (33, 34), and the most popular topics in concussion focus on the relationship between acute concussion and mental health outcomes rather than PPCS (35, 36). While studies are emerging that focus on PPCS, they predominantly report on interventions, treatments, and management (37–39). Therefore, the focus of this scoping review is to explore: (1) the mental health outcomes of individuals with PPCS and the types of assessments used to identify mental health outcomes this group, and (2) how mental health outcomes compare in terms of similarities and differences among pediatric and adult populations with PPCS. Specifically, we wanted to answer the question: *What are the associations of mental health outcomes and PPCS post-injury and types of assessments used that examine mental health outcomes across the lifespan in individuals with PPCS*?

## Methods

A scoping review was used to address study objectives. The purpose of a scoping review is to establish and survey the current field of literature on a given topic, identify common and key characteristics of studies or related concepts, and divulge existing gaps (40, 41), rather than comment on the quality of studies or confirm current practices and/or interventions (41), as a systematic review would do. A scoping review is preferential to a systematic review for this topic, as studies on mental health outcomes across the lifespan in individuals with PPCS has yet to be synthesized. Thus, a broad scope of the literature is warranted prior to a systematic review. Scoping review guidelines and frameworks were adopted in line with Arksey and O'Malley (42), with considerations from Levac et al. (40), as well as adhering to the PRISMA-ScR checklist (43).

### Stage 1: Identify the Research Question

Our goal was to explore the mental health outcomes that occur across the lifespan in those with PPCS. As PPCS has various terminology, definitions were set in order to thoroughly capture all studies that may mention any synonym of PPCS. PPCS was defined as any concussion disorder lasting longer than 4 weeks, and could be addressed as “PPCS,” “persistent concussion symptom (PCS),” or “chronic,” “long-term,” “persistent,” or “prolonged” followed by “mTBI,” “concussion,” “minor TBI,” or “mild/minor head injury.” As there is ambiguity in specific diagnostic criteria of PPCS, a minimum of 4 weeks was the chosen symptom duration. This criterion is also consistent with what is found in more recent literature (44, 45). There was no upper limit set on duration, to try and capture as many studies with persistent symptoms as possible.

For the purpose of our study, the term “adolescents” is defined as individuals aged 10–18 years old, according to the World Health Organization (46); and those aged younger than 10 years are described as “children.” For this review, the age range for pediatrics was operationalized to include children and adolescents aged 0–18 years old, but could be in their 19th year (e.g., 18 years, 11 months). To note, anyone in the children or adolescent range will be referred to hereinafter under the umbrella term “pediatric” to encompass this age span. Adults are defined as individuals aged 19 years and above. Any studies including “university-aged” participants with no stated age range, fell under the adult definition, as most university students are 19 years or older. Furthermore, mental health outcomes were defined as mood disorders or emotional outcomes that could be measured and assessed by standardized psychological tests or psychological screening measures, that have been reported as symptoms of PPCS. Mental health outcomes did not need to be clinically diagnosed, and terminology included but was not limited to “depression,” “anxiety,” “worry,” “sadness,” “aggression,” and “irritability.” Inclusion and exclusion of particular mental health outcomes will be addressed in the eligibility criteria. Moving forward for simplicity, all mTBI terminology will be referred to as “concussion,” and PPCS terminology referred to as “PPCS.”

### Stage 2: Identifying Relevant Studies

#### Eligibility Criteria

To be considered an eligible study, the participants in the study must have experienced a concussion, as classified by diagnostic measures, such as: ICD-10 criteria, Glasgow Coma Scale score between 13 and 15, loss of consciousness <30 min, and/or post-traumatic amnesia lasting <24 h (47). In addition, participants needed to experience at least one concussion symptom, classified by a concussion assessment measure such as the Post-Concussion Symptom Inventory (48), Post-Concussion Symptom Scale (49), or the Sport Concussion Assessment Tool (50), lasting for at least 4 weeks in duration. Concussions determined by self-report and healthcare professional were both included if definition criteria were met. All age groups were considered eligible, including infants and older adults. Injuries categorized as an acute concussion (<4 weeks post-injury), or moderate or severe TBI were excluded. Participants could have multiple previous concussions documented, but the primary focus of the study had to center on PPCS. Additionally, head injuries (including moderate and severe TBI) that were not related to their concussion (e.g., contusion, edema, hematoma, hemorrhages, fractures, or chronic traumatic brain injury) were also not included in the review. Studies with mental health outcomes including psychiatric/psychological disorders of anxiety (including generalized anxiety disorder, social anxiety, and phobias) and depression (major depressive disorder, and persistent depressive disorder) were eligible. Studies were not excluded if they did not include data on pre-injury personal or family psychiatric history, as post-injury data was the primary focus. Literature suggests that emotional and behavioral challenges are part of PPCS sequelae (51–53). Hence, we included studies that address commonly reported emotional symptoms that do not require a formal diagnosis. This includes sadness, worry, anger, irritability, and low mood. Of note, attention was excluded if it was classified as a cognitive domain. Quality of life studies were included, but had to explicitly measure an emotional outcome stated from this study (e.g., sadness, worry, etc.).

Studies were excluded if they were published prior to 1995, as this was the point at which PPCS emerged as accepted terminology, and extending the timeline prior to 1995 would make it difficult to distinguish what was captured as PPCS (54). Of note, the earliest study extracted from the search results was published in 2003. Studies could not be written in anything other than the English language. Further, only peer-reviewed primary studies were accepted, of both qualitative and quantitative nature. Study designs were limited to human subjects only. Studies could include a neuroimaging component, but they also required mental health outcome measures, rather than solely neuroimaging.

#### Database Search

Identifying studies relevant to the question of interest was achieved using four databases: Ovid MEDLINE, PsycInfo, CINAHL, and EMBASE. A hand-search of all reference lists from included studies was conducted to ensure relevant papers were captured in the search.

#### Search Strategy

Search queries and strategies used followed the population, concept, context framework (see Supplementary Tables 1–4 for database search strategies) (41). Each search was guided using Boolean operators “AND” and “OR” to create a relevant search queue. The appropriate truncations and adjacencies associated with each database were used, as well as MeSH terms and keywords, where applicable.

### Stage 3: Study Selection

In total, 20,731 studies were extracted from the four databases. Upon retrieval of all relevant papers from each database, EndNote X9®, a reference management software, was used to compile all papers, and remove duplicates. Once duplicates were removed, all papers were transferred to Covidence®, an online tool designed to streamline review processes, to begin screening the papers. A final number of 11,920 papers advanced to screening following de-duplication. Of note, an original database search was conducted in July 2020. A second, updated search was run from July 2020 to February 2021 due to the advancing literature in this subject area. All statistics and results have been updated to reflect the most recent search date.

Two screening processes were completed to ensure a detailed and thorough review of all relevant papers was practiced: (1) title and abstract screening, and (2) full text screening. Two independent reviewers completed title and abstract screening [ES, HA], with the same inclusion and exclusion criteria. The reviewers met prior to screening to discuss their strategy and ensure they were following the same criteria. A third, independent reviewer [BL] assisted with all stages of screening, charting, and analysis, as well as resolution of screening conflicts between the first and second reviewers.

**Figure 1:**
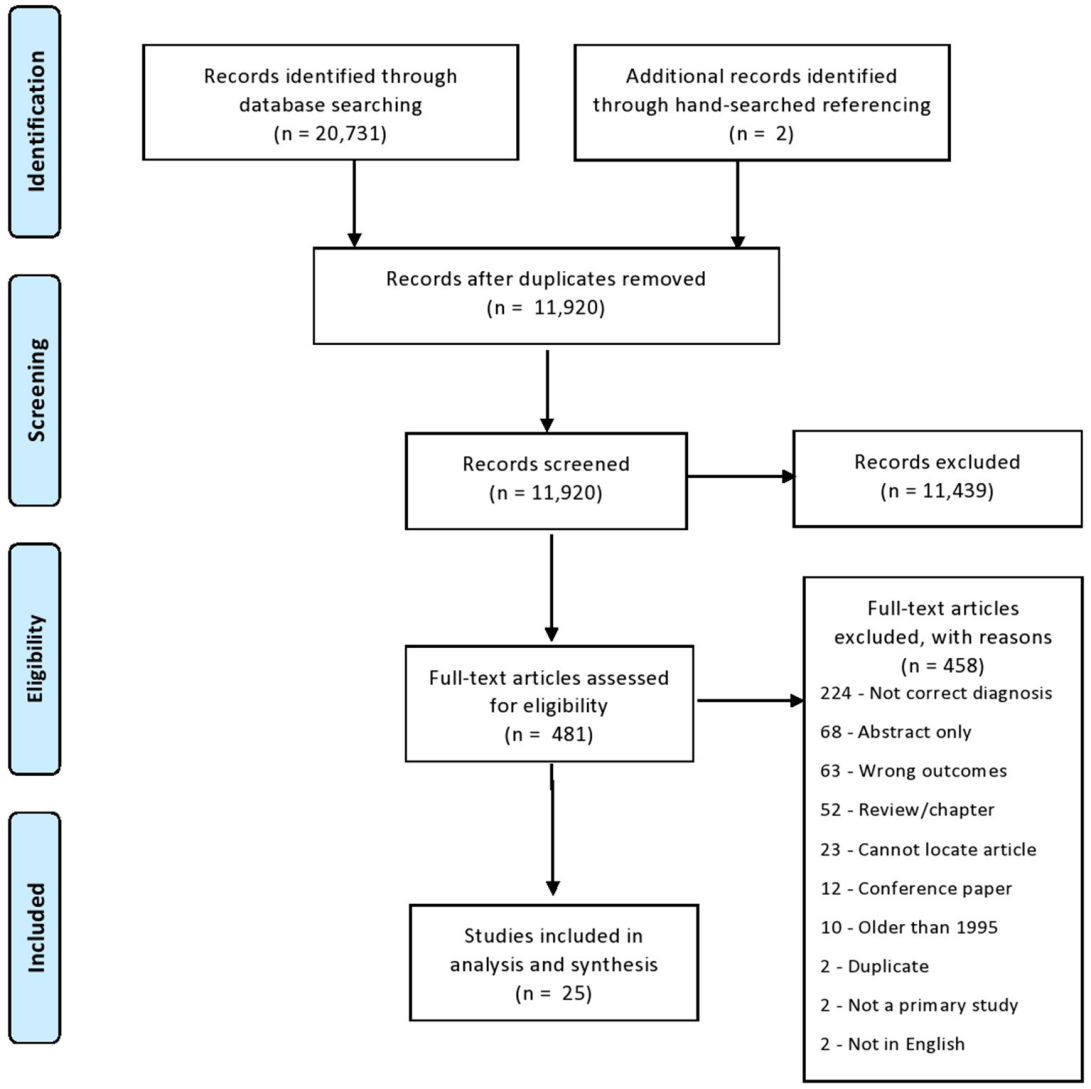
PRISMA flow diagram.

After title and abstract screening, 481 papers advanced to full text screening, also completed in Covidence®. Once included papers were finalized, papers were transferred to Mendeley®, another reference management tool, to house the full-text, and subsequently back to Covidence® to read the full text and screen. From full-text screening, 23 papers successfully met inclusion criteria. Additionally, two papers met inclusion criteria from hand-searched referencing, which arose from manually checking the reference lists of each included paper. Thus, a total of 25 papers were included in the scoping review. Four hundred and fifty-eight papers were excluded from review after full-text screening (see Figure 1 for full PRISMA diagram and exclusion justification).

### Stage 4: Charting the Data

Once all included studies were established, data was documented using Microsoft Excel® charting *via* spreadsheet program. The chart was co-created by all three reviewers and discussed thoroughly to ensure that all relevant and important details from each study were documented. Prior to charting all data, 10 studies were selected, reviewed and charted by all reviewers to serve as a pilot run. This ensured that reviewers were consistent in their documentation processes. The three reviewers met after pilot charting to review the process and study eligibility criteria.

General study characteristics (title, author, year, design, and objectives) were charted to archive traditional study qualities. Furthermore, following the “population, concept, and context framework” (40), population (participant group, gender, age, and sample number), concept (mental health outcome, assessment measurement, symptom severity, and key findings), and context (PPCS injury mechanism and history, terminology, outcome measurement, diagnosis information, and key findings) information were documented to chronicle important features, and draw comparisons and generate themes later during analyses (see Supplementary Table 5 for extraction chart template).

### Stage 5: Collating, Summarizing, and Analyzing Results

Following charting of all relevant data, Microsoft Excel® spreadsheets were used to collate results, resulting in summary tables, organized, and separated by pediatric and adult study findings. Each pediatric and adult summary table consisted of the following headings: author, year, study purpose, population (sample size and mean age), study design, mental health outcome and measurement, and key findings. Numerical analyses were used to understand the extent, breadth, nature, and distribution of included studies by evaluating the general study characteristics across papers (41). Furthermore, a smaller table was created to document the general mental health outcome findings within and between pediatric and adult studies, in order to better conceptualize the distribution of outcomes across groups.

## Results

Results are organized first according to general participant and study characteristics, followed by outcome measures (see Supplementary Table 5). Thereafter, mental health outcome results are summarized, and organized according to anxiety, depression, anxiety and depression, and “other” mental health outcomes, and further sub-categorized into pediatric and adult findings, to allow for comparisons between groups (see Table 1 for summary of outcome and assessments and Table 2 for summary of key findings across populations, and Supplementary Tables 6, 7 for detailed results of the studies).

**Table 1:** Outcome and assessment.

**Population**	**References**	**Sample size**	**Measure**	**Clinical problem of interest (outcome)**
Pediatric (0–19 years)	(26)	179	State trait anxiety inventory for children	Anxiety
	(55)	4,380	Post-concussion symptom scale	
	(56)	92	Children's depression inventory 2	Depression
	(57)	49	Patient health questionnaire-9	
	(58)	174	Interview to determine symptoms	Anxiety and depression
	(59)	48	Behavior assessment system for children, 2nd edition	
Adult (19+ years)	(60)	57	Anxiety item back from the quality of life in neurological disorder computerized adaptive tests	Anxiety
	(12)	343	Hospital anxiety and depression scale	
	(61)	1,229	Adapted version of the composite international diagnostic interview screening scales	Depression
	(62)	5	Qualitative interviews	
	(63)	190	British Columbia major depression inventory, 2nd edition	
	(64)	173	Center for epidemiological studies depression scale	
	(65)	67	Beck depression inventory, 2nd edition	
	(66)	213	Beck depression inventory, 2nd edition	
	(67)	170	General health questionnaire	
	(68)	126	Beck depression inventory, 2nd edition	
	(69)	163	Anxiety: Impact of event scale	Anxiety and depression
			Depression: Rivermead Post-concussion symptoms questionnaire	
	(70)	4,462	Hospital anxiety and depression scale	
	(71)	105	Depression and anxiety stress scales-42	
	(72)	162	Hospital anxiety and depression scale	
	(73)	72	Anxiety: Beck anxiety inventory, 2nd edition	
			Depression: Beck depression inventory, 2nd edition	
	(74)	80	Interviews with symptoms according to DSM-IV criteria	
	(75)	94	Hospital anxiety and depression scale	
	(76)	341	Hospital anxiety and depression scale	
	(77)	155	Glasgow outcome scale—extended	
	(69)	163	Impact of event scale (stress)	“Other”
	(62)	5	Qualitative interviews	

**Table 2:** Key findings across the lifespan.

**Clinical problem of interest**		**Pediatric populations**	**Adult populations**
Anxiety	*k* (%)	2 (8%)	2 (8%)
	Key findings	• Score distributions were significantly worse on the state trait anxiety inventory for children among participants with delayed symptom resolution compared with those with early symptom resolution (26) • Prolonged recovery group had significantly higher scores on all five symptom profiles, including anxiety (*p* < 0.001), than the typical recovery group. Older age was a major factor in the anxiety group (78)	• 14.9% of participants scored >7 on HADS anxiety scale, indicating clinically significant anxiety symptoms at follow-up, where 7.2% scored as mild, 5.6% as moderate, and 2.1% as severe (12) • Anxiety was statistically significantly correlated with PCS after adjusting for cluster, whereby higher anxiety was associated with greater reporting of persistent novel post-injury PPCS at follow up (12) • Total effect of anxiety of PPCS were significant, suggesting a direct effect of anxiety on PPCS through pain catastrophizing and through limiting behavior (58) • Anxiety strongly correlated with post-concussion symptoms (58)
Depression	*k* (%)	2 (8%)	8 (32%)
	Key findings	• Participants with higher PPCS tended to have higher levels of depressive symptoms; Decreases in PPCS were strongly related to decreases in depressive symptoms (79) • Significant correlation between depression scores and the number of concussion symptoms reported on the PCSI-Time I and Time 3 indicating a linear relationship (80) • High depression score increased by a factor of 11 if the child was admitted to hospital compared to those not admitted (80)	• Soldiers with high deployment stress exposure had significantly greater PPCS, post-traumatic stress, and depression factor scores (60) • Patients who experience MTBIs and who have a post-injury recovery course complicated by significant depression report more post-concussion symptoms, and more severe symptoms, than (a) outpatients with depression, and (b) patients with MTBIs who do not have significant symptoms of depression (81) • Patient group endorsed significantly higher levels of depression than controls (82) • 56 subjects (85%) had a psychiatric diagnosis, which is a considerable increase over 54% preinjury lifetime psychiatric diagnoses; BDI and PCS symptoms were significantly correlated (83) • Veterans with PPCS had more symptoms of depression than non-TBI veterans (64) • Patients who met ICD-10 criteria for PPCS had significantly higher BDI-II subscale total scores than patients who did not meet ICD-10 criteria at one month based on “mild or greater” symptom reporting and “moderate or greater” symptom reporting (65) • Subjects with depression reported higher levels of PCS even excluding symptoms that overlap with major depression (66)
Anxiety and Depression	*k* (%)	2 (8%)	9 (36%)
	Key findings	• 20/174 (11.5%) of patients met study criteria for a post-injury psychiatric disorder, including 14 patients diagnosed with a novel psychiatric disorder, 2 diagnosed with novel and isolated suicide ideation, and 4 who experienced worsening subjective symptoms of a pre-existing psychiatric disorder (57) • Significant findings on self-report measures for socioemotional perceptions, with concussion group having significantly higher T scores on internalizing symptoms composite (atypicality, locus of control, social stress, anxiety, depression) and emotional symptoms index (anxiety, social stress, depression, sense of inadequacy, self-reliance, and self-esteem subscales) relative to controls (56)	• Higher prevalence of depression was found in patients with an incomplete recovery (20%) as compared to patients with complete recovery (7%) (84) • Patients who developed PPCS reported higher of anxiety than the recovered group, and also experienced higher levels of depression than the recovered group (67) • One of the most common symptoms was depression (47.2%); women had a higher prevalence of depression than men (75) • 50% of mTBI veterans were diagnosed with generalized anxiety disorder; 57% of mTBI veterans were diagnosed with major depressive disorder (84) • 37 (35.2%) of patients had clinically elevated symptoms of depression, anxiety, or both (clinically elevated beyond the normal range) (69) • PPCS group had higher total scores on BDI-II and BAI compared with no PPCS (85) • Depression after injury = 35 (44%); Anxiety after injury = 19 (24%); Total psychiatric conditions = 60 (76%) (86) • New conditions that developed post-injury, and had a two–three-fold increase in the prevalence of psychiatric conditions after injury, most increase took form of anxiety and depression (86) • The psychosocial symptoms, such as depression and irritability, were significant at the late after injury stage (4 and 8 weeks) (87)
“Other”	*k* (%)	0 (0%)	2 (8%)
	Key findings		• Heightened emotions such as frustration, mood swings, depression, suicidal thoughts, and changes in behavior (88) • Moderate to severe stress reported in 10% of men and 14% of women; Women reported significantly higher depression scores and stress scores (75) • Mental limitations stemming from the injury such as irritability and anger (75)
Total		6 (24%)	19 (76%)

### General Participant and Study Characteristics

Of the 25 studies published from 2003 to 2021, six studies focused on children and adolescents, and the remaining 19 focused on adults. The primary location of studies was within North America, originating in the United States (*n* = 11) or Canada (*n* = 7), followed by a small number in Europe, including Sweden (*n* = 2), Finland (*n* = 1), and The Netherlands (*n* = 1). The remaining studies were in New Zealand (*n* = 1), Australia (*n* = 1), and China (*n* = 1). Sample size was broad amongst studies, with as small as five participants, to as large as 4,462 participants. The varied distribution of sample sizes is displayed in Table 1. There was heterogeneity in the distribution of male and female representation across studies; the majority had a relatively equal proportion, where the male-female ratio fell between 40 and 50% (*n* = 13); however, when comparing unequal distributions, there was much greater number of studies with male majority (*n* = 9) than female majority (*n* = 3). Age of participants ranged from 8 to 19 years in pediatric studies, and 19–91 years in adult groups. The primary participant group featured outpatients from past hospital visit (e.g., emergency department) or TBI clinics (*n* = 13). Other studies included pediatrics with past unspecified or sports-related concussion (*n* = 4), army or combat veterans (*n* = 3), community-dwelling participants (*n* = 3), university students (*n* = 1) or older adult populations (*n* =1). Nineteen studies required a formal diagnosis of concussion (e.g., by physician or nurse practitioner) and five relied on self-report. One study did not specify how they were diagnosed. Furthermore, while seven studies included pre-injury personal and/or family mental health history in their data but was not the main outcome of the study, and many studies did not document or assess pre-injury mental health personal or family history (*n* = 14).

Most studies were quantitative in nature, using a cohort (*n* = 10), cross-sectional (*n* = 4), or case-control/case-series (*n* = 4) design. Other quantitative studies included descriptive analysis (*n* = 2), retrospective chart review (*n* = 1), a clustered randomized trial (*n* = 1), and a randomized controlled trial (*n* = 1). There was also one qualitative, interpretative phenomenological study, and one mixed-methods study. While we were not interested in intervention studies, the designs utilizing a trial method included follow-up data from an intervention study, rather than the trial itself (12, 54). Cohort designs typically compared participants with PPCS to healthy controls, or to participants with resolved or acute concussion. Some studies also compared PPCS participants who also had a mental health outcome, such as symptoms of depression, to PPCS participants without psychiatric symptoms or diagnoses. Terminology related to PPCS fell under a large umbrella of terms but were based on length of symptom duration criteria; almost all studies referred to PPCS as persistent or long-term post-concussion or post-concussive symptoms or syndrome, in conjunction with concussion; other less common terms were prolonged, protracted, or persistent concussion, as well as persistent symptoms after a concussion.

### Outcome Measures

Due to the variety of outcome measures used across studies, between different mental health outcomes, and varying assessments based on age range, the measures used are important to present. Across the six pediatric studies, six different measures were utilized to assess the clinical problem of interest. Anxiety measures included the State-Trait Anxiety Inventory for Children (26, 78), and the Post-Concussion Symptom Scale (55, 79, 89). Depressive symptom measures consisted of the Children's Depression Inventory (57, 80) and the Patient Health Questionnaire-9 (56, 90). Combined symptoms of anxiety and depression studies utilized a semi-structured interview (58) or the Behavior Assessment System for Children, 2nd Edition (59, 91).

Outcome measures utilized for adults were also quite varied across studies. The two anxiety studies utilized two different measures; the anxiety item bank from the Quality of Life in Neurological Disorder Computerized Adaptive Tests (60, 92), and the Hospital and Anxiety Depression Scale (12, 93). The eight adult studies that centered on symptoms of depression utilized the adapted version of the Composite International Diagnostic Interview Screening Scales (61, 88), qualitative interviews (62, 81), the British Columbia Major Depression Inventory, 2nd Edition (63, 82), the Center for Epidemiological Studies Depression Scale (64, 83), the Beck Depression Inventory (65, 66, 68, 94), or the General Health Questionnaire (67, 84). Between the nine adult studies on combined symptoms of anxiety and depression, four used the Hospital Anxiety and Depression Scale (70, 72, 75, 76, 93), while others used the Impact of Event Scale (69, 95), the Rivermead Post-Concussion Symptoms Questionnaire (69, 96), Depression and Anxiety Stress Scales-42 (71, 85), the Beck Anxiety Inventory (73, 86) and Beck Depression Inventory (73, 94), interviews with symptoms according to DSM-IV criteria (74, 87), and the Glasgow Outcome Scale -Extended (77, 97). Finally, the two adult studies that included “other” mental health outcomes, included the Impact of Event Scale (69, 95) and qualitative interviews (62, 81).

### Anxiety

#### Pediatric

Two studies measured anxiety outcomes among children and adolescents with PPCS (26, 55). The first study characterized the psychological factors associated with persistent symptoms following a concussion in pediatric participants (aged 8–18 years old). The second study examined the difference of symptom burden between pediatric participants (aged 10–18 years) with prolonged recovery to those with typical recovery (55). Both studies demonstrated anxiety symptoms were significantly worse among participants with delayed symptom resolution compared to those with early symptom resolution (26, 55). In particular, Schilling et al. (55) demonstrated there was a higher score on the anxiety profile in the prolonged recovery group than the typical recovery group, indicating higher severity and symptom burden.

#### Adults

Two studies measured anxiety outcomes among adults with PPCS (12, 60). One study (60) tested the strength of the cognitive and behavioral pathways that mediate the relationship between anxiety and post-concussion symptoms among patients with concussion. The other study (12) examined PPCS and anxiety in patients with concussion. Results demonstrate that anxiety could be correlated with PPCS, and in particular, clinically significant anxiety (HADS-anxiety score > 7) was reported by 12.8% and significantly associated with symptom reporting in patients (12).

### Depression

#### Pediatric

Of the six studies that centered around pediatric populations with PPCS, only two studies measured symptoms of depression (56, 57). Overall, both studies reported that increased PPCS symptoms were correlated with increased symptoms of depression, especially closer to the time of the injury. The first study (57) followed pediatric participants (11–17-years-old) with prolonged recovery following sports-related concussion and found that participants with higher symptom severity of PPCS also reported higher symptoms of depression. As well, participants who had higher initial symptoms of depression and PPCS (onset of or increased intensity in three or more post-concussive symptoms for at least 1 month duration following injury) significantly predicted a larger decline in symptoms over time, indicating an improvement in symptoms of depression for these subgroups. The second study (56) measured symptoms of depression in pediatric participants (8–18 years-old) to investigate predictors of developing depression in individuals with persistent concussion symptoms. This study found symptoms of depression in 22% of participants and noted that this group had significantly higher PPCS symptom severity scores than non-depressed participants. Interestingly, the adolescent depression scores increased by a factor of 11 if they also had a hospital admission as a result of concussion.

#### Adults

Of the 19 studies that centered around adults with PPCS, eight studies measured symptoms of depression (61–68). Like in the pediatric population, most studies found that participants reporting higher PPCS symptoms also reported higher severity of depression symptoms (64, 65, 67, 68, 82). For example, 61% of participants with PPCS reported presence of a depressive disorder, endorsing elevated symptoms using self-reported measures (65). Also noted in two studies, symptoms of depression was present in participants with PPCS, even without including overlapping symptoms between PPCS, PTSD, and depression, such as depressed mood, insomnia, fatigue, irritability, restlessness (65, 67). In a study with a participant group of veterans, it was reported that deployment stress was related to an increase in both PPCS scores and depression scores (61). Furthermore, in a qualitative study conducted with university students, all five participants reported symptoms of depression following PPCS, and one participant described a previous suicide attempt following concussion (62).

### Anxiety and Depression

#### Pediatric

There were two studies that looked at anxiety and depression (58, 59) in the pediatric population. Both studies reported increased symptoms of depression in individuals with PPCS. The first study by Ellis et al. (58) looked at psychiatric diagnoses following a sports-related concussion. Of the pediatric participants (19-years-old or younger), 14 (8.0%) were diagnosed with a novel psychiatric disorder. Two (1.1%) participants reported suicidal ideation and 4 (2.3%) experienced worsening symptoms of a pre-existing psychiatric disorder. Moreover, 10 (52.6%) individuals with novel psychiatric disorder were diagnosed with a depressive disorder, 4 (21.1%) with a new-onset anxiety disorder, and 1 (5.3%) with major depressive disorder and an anxiety disorder. The second study (59) measured the severity of anxiety and depression symptoms in 48 pediatric participants (13–17-years-old), in which those with a history of concussion reported significantly higher anxiety and depression symptoms.

#### Adults

There were nine included studies that analyzed both anxiety and depression on in an adult sample (69–77). For each of these studies, the severity of both depression and anxiety was measured. There were five studies that reported a higher prevalence of anxiety and depression symptoms in patients who develop PPCS (69, 71, 72, 74, 75). Four studies did not find a significant difference in both outcomes (69, 73, 77, 98). Two studies reported higher depression scores in those with PPCS, however had no significant differences in anxiety scores when compared with the general population (74, 77). Moreover, one study found the prevalence of anxiety and depression symptoms to be comparable to the general population (76). Although Doroszkiewicz et al. (71) found no statistical significance between any variables (i.e., sex, gender, age, and number of concussion) and PPCS, they found that individuals with PPCS reporting higher anxiety and depression symptoms had lower quality of life scores, as measured by the World Health Organization Quality of Life Assessment-BREF (99).

### “Other” Mental Health Outcomes

#### Pediatrics

No studies that met our inclusion criteria examined psychological outcomes beyond symptoms of anxiety and depression in pediatric populations.

#### Adults

There were two studies that measured other emotional or behavioral outcomes among adults (62, 69). The first study (62) looked at mood swings, heightened emotions, and suicide ideation as mental health outcomes. This study used interpretative phenomenological analysis (81) to explore the experiences of five female university athlete's post-concussion. In several participants, there were more feelings of frustration and changes in mood 1-month post-concussion. One participant reported a suicide attempt and extreme emotional distress (62). The second study (69) characterized the long-term consequences of concussion and post-concussion symptoms in men and women. One of the outcomes included stress reactions, which found that women reported higher scores compared to men. Moderate to severe stress was reported by 10% of men and 14% of women with PPCS.

## Discussion

This scoping review reported a total of 25 papers relevant to our question: *What are the associations of mental health outcomes and PPCS post-injury and types of assessments used that examine mental health outcomes across the lifespan in individuals with PPCS*? Furthermore, we investigated the differences between the six pediatric studies, in comparison to the 19 adult studies. In general, these studies largely focused on symptoms of anxiety and depression with minimal coverage on other emotional outcomes common to PPCS such as stress, irritability, aggression, or frustration. The common trend shown amongst studies was a higher association of symptoms of anxiety, symptoms of depression, or “other” mood disorders in those with higher PPCS scores. While this was not representative of all studies, most studies reported statistical significance of higher psychiatric symptoms correlated with PPCS groups. This was compared to normative data in most studies, but six did include typically developing (*n* = 5) or orthopedic injury (*n* = 1) control groups. In pediatric settings, two of the six studies found significantly increased mood symptoms (depression: *n* = 1, anxiety and depression: *n* = 1), as compared to normative data or a typically developing control group. This ranged from 22% for depression studies (57) and 11.5% for anxiety and depression studies (58). The remaining four of the six studies did not include results related to prevalence, instead commenting on the significance of relationship between PPCS and mental health outcomes (anxiety: *n* = 2, depression: *n* = 1, anxiety and depression: *n* = 1).

In adult populations, all but one study (*n* = 18) reported that increased PPCS symptoms related to increased mood symptoms (anxiety: *n* = 2, depression: *n* = 8, anxiety and depression: *n* = 8, “other”: *n* = 2). Prevalence ranged from as high as 44% of PPCS participants with increased depression symptoms (77), as compared to as high as 24% of PPCS participants with increased anxiety (74). In studies that combined symptoms of anxiety and depression, results were staggering, reporting a prevalence as high as 76% in individuals with PPCS (74). The remaining study (76) concluded no differences between those with PPCS and the general population.

In general, the findings also suggest that research specific to certain PPCS age groups is lacking, noted in both young pediatric and older adult settings. While eligibility criteria were inclusive of all ages, no pediatric participants were younger than 8 years old. Concussions in infants and young children are widely underreported and understudied, even though they are a high-risk incident group (98, 100, 101). Furthermore, only six of the 25 studies focused on children and adolescents. This is of concern as incidence rates of pediatric concussion are increasing, and likely at a higher rate than reported due to under-reporting of concussion by adolescents, parents, and coaches (102, 103). This leads to apprehension about whether these studies are truly representative samples that reflect prevalence in the population. Children and youth are at a critical period where mental health is impressionable and malleable, justifying the need for more pediatric studies. In addition, only one study focused on geriatric populations (72), even though falls are a primary cause of concussion, and common in older adults (104, 105). Furthermore, older adults report more pre-existing cognitive impairments that make it difficult to treat and manage concussion (106), as well literature suggests older adults can experience poor quality of life post-concussion, due to factors such as reduced independence and difficulty in recovery due to comorbidities or frailty (107). As most studies (17/25) focused exclusively on individuals aged 19–60 years old, there is a need to conduct more studies that target pediatric and older adult populations in these critical life stages.

Furthermore, most papers measured symptoms of depression (10/25), or symptoms of anxiety and depression together (10/25). Upon analysis of findings, there was a clear distinction between symptoms of anxiety, symptoms of depression, and what we categorized as “other” mental health outcomes. Results demonstrate fewer papers on anxiety and PPCS. Hence, future research should focus on anxiety-based outcomes since it is highly reported in adolescents and adults with PPCS (104). As there is ambiguity among distinguishable features of PPCS and mental health disorders such as symptoms of anxiety and depression, further interest on anxiety onset post-concussion is also necessary to help identify mental health outcomes as potential diagnostic symptoms of PPCS. Additionally, almost no studies investigated mental health outcomes or symptoms other than depression and anxiety. Emotional symptoms such as stress, irritability, and aggression were reported in adolescents and adults with PPCS (108). The Rivermead Post-Concussion Symptoms Questionnaire, a commonly used diagnostic assessment for PPCS, includes mood disturbances such as irritability and frustration, despite minimal literature and investigation on these symptoms in adolescents and adults (97, 109). Thus, more research is needed in this area to understand the impact of mood disturbances beyond depression and anxiety following PPCS. Related, the reporting of PPCS itself may include symptom overlap with mental health outcomes like depression, such as fatigue, low mood, and irritability (110). Few studies made distinctions between defining features of depression that overlap less with PPCS, and is important to consider in future studies moving forward.

In addition, only a small number of studies included prior mental health diagnosis and/or family history in their demographic information and/or analyses. Causal associations of mental health in those with concussion and PPCS, including symptoms of anxiety and depression, remain arguable and inconclusive. Many studies show evidence of an increase in the severity of mental health symptoms in those with PPCS, emphasized and exacerbated if those individuals had pre-existing psychiatric symptoms prior to injury (68, 111). Family and personal psychiatric history are of notable causal interest of PPCS symptoms as a retrospective cohort study of concussed high school athletes were 5 times more likely to develop PPCS with family or personal pre-existing psychiatric symptoms (31). Strikingly, a study by Iverson (110) found that ~9 out of 10 individuals diagnosed with depression met self-report criteria for PPCS, without concussion history. Not only do studies suggest mental health history may play a contributing role in severity of PPCS, but multiple conditions could be a factor, including chronic pain (112) and cerebrovascular injuries (113). Contrastingly, other studies support findings of presence of mental health symptoms post-concussion, in those without any prior history of mental health (68, 77, 83, 114). While documentation of prior mental health history was not a requirement for study inclusion, it does provide insightful knowledge on the question of causality of prolonged emotional and behavioral symptoms of PPCS for future studies.

The number of methods, measures, control groups, and samples sizes used to assess both mental health outcomes and PPCS criteria in individuals was extremely diverse. In fact, there were four pediatric-specific measures and twenty measures inclusive of adult and/or pediatric populations used to capture mood throughout the reviewed studies. The impact of method heterogeneity is two-fold: diversity can create more avenues to explore factors related to mental health outcomes that may otherwise be overlooked but can also create a lack of consistency in assessments, leading to inconclusiveness and skepticism of study quality. Additionally, broad differences amongst study designs, control groups, and sample sizes augment the conversation regarding variables that can create discrepant results amongst studies and impact subsequent findings. These findings of heterogeneity supports the call for common data elements in TBI research, such as those outlined by the National Institute of Neurological Disorders and Stroke, which aims to standardize and unify data collection studies related to TBI to increase efficiency and efficacy (115, 116).

Almost all studies used quantitative designs such as observational cohort, case-control studies, or randomized trials. Only one included study used a qualitative design to document the thoughts and feelings of university-aged students suffering from PPCS (81). Using in-depth semi-structured interviews, findings reported on extremely personal details, such as suicidal ideation and attempts post-injury. Incorporating qualitative and mixed methods for data collection could offer in-depth narratives and complex emotions that quantitative studies fail to capture. Qualitative studies on lived experiences following PPCS exist to broadly magnify quality of life rather than emotional and social domains (117, 118), and literature on emotional and social aspects of PPCS are beginning to emerge slowly. For example, a study by Davies et al. (51) was published recently on the emotional and social burden of persistent concussion. To note, while this study reported looking at persistent concussion, they did not specify the duration of symptoms. Thus, it did not fit eligibility criteria.

Finally, our definition of PPCS was created based on the current literature. However, there are varying definitions of PPCS, and ambiguity of criteria exists around included symptoms and severity, duration of symptoms, and diagnostic tools. Current definitions of PPCS are not specific to concussion (119). There are several explanations for the heterogeneity in PPCS definitions, one of which was most recently applied to the predisposing, precipitating, and perpetuating (PPP) model. The PPP model proposes that factors contributing to the course and development of PPCS are individualistic, and must consider personal attributions (120). While it is understandable that PPCS duration and symptoms are variable due to individual and personal factors, it does not make it easier to form a consensus on definitions and criteria, or to gather all pertinent literature on the subject matter. The most recent uniform definition for PPCS emerged from experts using the Delphi Method, which was created on the basis of three criteria: (1) symptoms must occur within hours of concussion, and continue for a minimum of 3 months consistently; (2) must not be symptoms present from a pre-existing condition, and (3) impacts at least one domain of everyday life (15). This Delphi study could suggest that a central body should try to create unity on this matter. Reference to these new studies is important as it speaks to the rapidly evolving nature of the concussion literature and interest in persistent presentations more broadly.

The natural progression of this review prompts a systematic review, to assess the diversity of study methods and designs, and to provide an overview of study quality and critical appraisal. Study evaluation will lead to greater understanding about what kinds of methods produce reliable and rigorous results, while also addressing designs that have the potential to produce meaningful results and could be employed further. Future meta-analysis is warranted to rigorously examine the strength of evidence provided in these studies, to comment on value, purpose, and effectiveness of the outcome assessments, study designs and methodologies.

### Strengths and Limitations

There are both strengths and limitations to the study processes that should be reflected on to improve future review processes. Chosen exclusion criteria may have limited relevant studies included in this review, as we chose to narrow our search to the English language (and translations), as well as limit the time frame to studies occurring in or after 1995. Furthermore, our choice of definition for PPCS may not have truly captured the varying terminology used across previously published literature, due to the inconclusiveness of the definition itself. Therefore, it is possible there are important studies missing. In addition, restricting the terms for mental health outcomes could have also decreased results, as we chose to focus on mood disturbances related to mental health disorders and PPCS, but did not specifically include many disorders themselves.

One strength of this study was having three reviewers engaged in all stages of the review process and having weekly meetings for transparency and constant communication. This added to the reliability of study results, as three people completed screening (title and abstract, and full text), charting the data, and analysis of findings. While this lengthened the duration of each step of the review, it strengthened the integrity of the studies chosen and reviewed. Furthermore, our study included literature from four established databases that created a broad and diverse landscape for possible studies included, from medical, psychological, and international sources.

### Implications

The findings of this study highlight the importance of interdisciplinary care for individuals with PPCS. Along with the cognitive and somatic symptoms that individuals may experience, symptoms such as anxiety and depression can be common. Moreover, the prevalence of anxiety and depression symptoms suggests that access to mental health interventions are important for individuals experiencing PPCS (58, 65, 70, 74). Thus, a biopsychosocial and holistic approach that addresses both physical and mental health needs should be emphasized in clinic and research settings (121). The complexities of PPCS warrant multiple healthcare professionals to provide specialized care that can encompass the range of symptoms they can experience—physically, mentally, and emotionally. The emotional burden associated with PPCS may also come with economic consequences. For example, the additional healthcare costs from mental health interventions may be a financial burden at the individual and population levels (122, 123).

This scoping review describes the various mental health outcomes that may emerge in those with PPCS. However, individuals with PPCS form a diverse group with different demographic characteristics (124) and mental health outcomes may differ based on these variables. For example, female sex is associated with greater frequency of PPCS (125). Further exploration of differences based on sex may serve as a prognostic indicator of poor mental health in this group. Future studies should also look at other socio-demographic variables that may predict mental health outcomes in this population. Psycho-social factors such as social support and family dynamics have been associated with PPCS frequency (126, 127), however, their contribution to mental health should be more closely examined. In addition, demographics of pre-existing personal and family mental health history should be carefully considered when designing, conducting, and analyzing studies. Given the diverse findings regarding PPCS and mental health outcomes, with both pre-existing mental health history and lack thereof reporting increase in psychiatric symptoms, there is a need to conduct further research exploration in this evolving area. This study included a review of the associations of mental health outcomes in individuals with PPCS and types of assessments used to capture these outcomes. This allowed us to survey different ways that mental health was assessed.

For many studies in this scoping review, PPCS is associated with an increased incidence of symptoms anxiety, symptoms of depression, and related mental health problems. However, the magnitude of this association is unknown. Since there appears to be a risk for mental health concerns after concussion, future research should quantify the strength of this relationship in those with PPCS. Obtaining prevalence estimates of anxiety and depressive disorders in those with PPCS may inform healthcare providers on the extent of this problem (24) and can support the development of optimal care strategies and pathways.

## Conclusion

Mental health outcomes are a primary concern following concussion especially in individuals experiencing persistent symptoms post-injury. Of particular interest, pediatric (or adolescent) and adult populations with PPCS exhibit symptoms of depression and anxiety. Furthermore, there is evidence to suggest that mood difficulties such as frustration, irritability, stress, attention, and worry can arise as a result of PPCS. However, minimal research has focused on this area. Both adolescents and adults suffer from PPCS, but literature on adolescents appears to be lacking compared to adults.

This scoping review highlights the existence of mental health outcomes following PPCS. It also outlines the need for further research in this area. For example, greater clarity and advancements should be made to reach a universal definition of PPCS. Also, it is important to investigate prevalence estimates in emotional and behavioral symptomatology among adolescents and adults with PPCS, with the lack of findings present. Emphasis should be directed toward studies involving children and adolescents, who are at a vulnerable age for cognitive, behavioral, and emotional development. As concussion becomes increasingly more common, it is important to advance clarity and insight for those experiencing prolonged and challenging symptoms.

## Author Contributions

ES and SS conceived the main idea and objectives of the review. ES, HA-H, and BL carried out all review steps, including search query, screening, analysis, charting of the data, and wrote the manuscript, with support from BG, AW, MB, BD, NR, and SS. NR and SS supervised the project. All authors provided critical feedback of drafts and contributed to the final version of the manuscript.

## Conflict of Interest

The authors declare that the research was conducted in the absence of any commercial or financial relationships that could be construed as a potential conflict of interest.

## Publisher's Note

All claims expressed in this article are solely those of the authors and do not necessarily represent those of their affiliated organizations, or those of the publisher, the editors and the reviewers. Any product that may be evaluated in this article, or claim that may be made by its manufacturer, is not guaranteed or endorsed by the publisher.
