# Supplementary material for: Mental Health Outcomes Across the Lifespan in Individuals With Persistent Post-Concussion Symptoms: A Scoping Review
Source: Front Neurol. 2022 Apr 11;13:850590. doi: 10.3389/fneur.2022.850590 (PMC9035995; doi:10.3389/fneur.2022.850590)
Supplement: Supplementary file 1 [file Table_1.DOCX]

Supplementary Material

**Table 1: Ovid MEDLINE Search Outline**

| # | Search Query | Results |
| --- | --- | --- |
| 1 | Post-Concussion Syndrome/ | 1177 |
| 2 | brain injuries, traumatic/ or brain concussion/ or brain injury, chronic/ | 16624 |
| 3 | (post-concuss* or post concuss* or postconcuss).tw,kf | 3204 |
| 4 | ((persist* or prolonged or chronic or long-term) adj3 (brain injur* or concuss* or head injur*)).tw,kf. | 41823 |
| 5 | (mtbi or mild trauma* brain injur* or mild TBI).tw,kf | 6278 |
| 6 | mood disorders/ or mental health/ or depressive disorder, major/ or anxiety disorder/ or depressive disorder/ | 177356 |
| 7 | ((mood or major depress* or anxi* or generalized anxiety*) adj3 (disorder* or syndrome* or outcome*)).tw,kf | 85554 |
| 8 | emotions/ or anger/ or anxiety/ or psychological distress/ or frustration/ or hostility/ or sadness/ or confusion/ or irritable mood/ or impulsive behaviour | 168456 |
| 9 | (emotion* or irritable or irritability or angry or anger or anxi* or distress* or frustrat* or hostil* or sad* or confus* or worr* or impulsiv* or attention*).tw,kf | 1073655 |
| 10 | 1 or 2 or 3 or 4 or 5 | 49523 |
| 11 | 6 or 7 or 8 or 9 | 1241287 |
| 12 | 10 and 11 | 6790 |

**Table 2: PsychInfo Search Outline**

| # | Search Query | Results |
| --- | --- | --- |
| 1 | brain injuries, traumatic/ or brain concussion/ or brain injury, chronic/ | 2415 |
| 2 | (post-concuss* or post concuss* or postconcuss).tw | 1080 |
| 3 | (mtbi or mild trauma* brain injur* or mild TBI).tw | 3781 |
| 4 | ((persist* or prolonged or chronic or long-term) adj3 (brain injur* or concuss* or head injur*)).tw | 971 |
| 5 | post-concussion syndrome.tw | 222 |
| 6 | mood disorders/ or mental health/ or depressive disorder, major/ or anxiety disorder/ or depressive disorder/ | 100656 |
| 7 | ((mood or major depress* or anxi* or generalized anxiety*) adj3 (disorder* or syndrome* or outcome*)).tw | 83221 |
| 8 | emotions/ or anger/ or anxiety/ or psychological distress/ or frustration/ or hostility/ or sadness/ or confusion/ or irritable mood/ or impulsive behaviour/ | 129155 |
| 9 | (emotion* or irritable or irritability or angry or anger or anxi* or distress* or frustrat* or hostil* or sad* or confus* or worr* or impulsiv* or attention*).tw | 907932 |
| 10 | 1 or 2 or 3 or 4 or 5 | 6116 |
| 11 | 6 or 7 or 8 or 9 | 994471 |
| 12 | affective disorders/ | 14539 |
| 13 | acting out/ | 851 |
| 14 | 11 or 12 or 13 | 995060 |
| 15 | 10 and 14 | 1799 |

**Table 3: CINAHL Search Outline**

| # | Search Query | Results |
| --- | --- | --- |
| 1 | (MH “Postconcussion Syndrome”) | 825 |
| 2 | (MH “Brain Concussion+”) | 5483 |
| 3 | T1 (mild TBI or mTBI or mild trauma* brain injur*) OR AB (mild TBI or mTBI or mild trauma* brain injur*) | 2132 |
| 4 | T1 (post-concuss* or postconcuss* or post concuss*) OR AB (post-concuss* or postconcuss* or post concuss*) | 1260 |
| 5 | T1 (chronic brain injur* OR AB chronic brain injur*) | 7 |
| 6 | T1 ((persist* or prolonged or chronic or long-term) N3 (brain injur* or concuss* or head injur*) OR AB ((persist* or prolonged or chronic or long-term) N3 (brain injur* or concuss* or head injur*)) | 867 |
| 7 | (MH “Anxiety+”) OR (MH “Anxiety Disorders+”) OR (MH “Generalized Anxiety Disorder”) | 89378 |
| 8 | (MH “Depression+”) | 117749 |
| 9 | (MH “Affective Disorders+”) | 124485 |
| 10 | (MH “Mental Health”) | 41253 |
| 11 | T1 ((mood or mental or affective or anxiet* or depress*) N3 (disorder* or syndrome* or outcome*)) OR AB ((mood or mental or affective or anxiet* or depress*) N3 (disorder* or syndrome* or outcome*)) | 54142 |
| 12 | (MH “Emotions+”) | 143730 |
| 13 | (MH “Sadness”) | 213 |
| 14 | (MH “Confusion”) | 2477 |
| 15 | (MH “Anger”) | 5744 |
| 16 | (MH “Acting Out”) | 146 |
| 17 | (MH “Attention+”) OR (MH “Attention Deficit Hyperactivity Disorder”) | 33168 |
| 18 | T1 (emotion* or irritab* or anger or angry or sad* or distress* or frustrat* or confus* or worr* or impuls* or attention or hostil*) OR AB (emotion* or irritab* or anger or angry or sad* or distress* or frustrat* or confus* or worr* or impuls* or attention or hostil*) | 275279 |
| 19 | 1 or 2 or 3 or 4 or 5 or 6 | 7546 |
| 20 | 7 or 8 or 9 or 10 or 11 or 12 or 13 or 14 or 15 or 16 or 17 or 18 | 546430 |
| 21 | 19 and 20 | 1548 |

**Table 4: EMBASE Search Outline**

| # | Search Query | Results |
| --- | --- | --- |
| 1 | exp post-concussion syndrome/ | 2518 |
| 2 | brain injuries, traumatic/ or brain concussion/ or brain injury, chronic/ | 71221 |
| 3 | (post-concuss* or post concuss* or postconcuss).tw,kw | 4798 |
| 4 | ((persist* or prolonged or chronic or long-term) adj3 (brain injur* or concuss* or head injur*)).tw,kw | 3047 |
| 5 | (mtbi or mild trauma* brain injur* or mild TBI).tw,kw | 9789 |
| 6 | mood disorders/ or mental health/ or depressive disorder, major/ or anxiety disorder/ or depressive disorder/ | 333740 |
| 7 | ((mood or major depress* or anxi* or generalized anxiety*) adj3 (disorder* or syndrome* or outcome*)).tw,kw | 123849 |
| 8 | emotions/ or anger/ or anxiety/ or psychological distress/ or frustration/ or hostility/ or sadness/ or confusion/ or irritable mood/ or impulsive behaviour | 441862 |
| 9 | (emotion* or irritable or irritability or angry or anger or anxi* or distress* or frustrat* or hostil* or sad* or confus* or worr* or impulsiv* or attention*).tw,kw | 1495517 |
| 10 | 1 or 2 or 3 or 4 or 5 | 80049 |
| 11 | 6 or 7 or 8 or 9 | 1833048 |
| 12 | 10 and 11 | 10594 |

**Table 5: Extraction Chart Template**

|  | General Study Characteristics | | | | | Population Characteristics | | | |
| --- | --- | --- | --- | --- | --- | --- | --- | --- | --- |
| Count | Author | Year | Title | Objectives | Design | Participant Group | Gender | Age (u, sd) | n |

| PPCS Characteristics | | | | | | Mental Health Characteristics | | |
| --- | --- | --- | --- | --- | --- | --- | --- | --- |
| Concussion terminology | Concussion history | Diagnosis definitions | Injury mechanism | Outcome (measurement, duration) | Key findings | Mood diagnosis/outcome | Assessment measure | Key findings |

**Table 6: Detailed Results Chart (Pediatric)**

| **Pediatric** | | | | | |
| --- | --- | --- | --- | --- | --- |
| Count | Author, year | Purpose | Population (n, mean age) | Design | Key Findings |
| 1 | Ellis, et al., 2015 | To examine the prevalence of emotional symptoms among children and adolescents with a sports-related concussion who were referred to a multidisciplinary pediatric concussion program  To examine the prevalence, clinical features, risk factors, and management of postinjury psychiatric outcomes among those in this clinical population | 174, 14.2 | Retrospective  chart review | - 20/174 (11.5%) of patients met study criteria for a postinjury psychiatric  disorder, include 14 patients diagnosed with a novel psychiatric disorder, 2 diagnosed with novel and isolated suicide ideation, and 4 who experienced worsening subjective symptoms of a pre-existing psychiatric disorder - Of patients who developed postinjury psych outcomes, 15 (75%) had 1 or 2 lifetime concussions - Psychosocial stress reported by 12 (60%) of patients who developed postinjury psych outcomes 19 NPDS were diagnosed among 14 patients, including depressive  disorders (n = 10, 52.6%), anxiety disorder (n = 4 (21.1%), MDD with secondary anxiety (n 1, 5.3%) - At time of postinjury psychiatric outcome assessment, 18 (90%) of 20 patients met ICD-10 criteria for PCS |
| 2 | Grubenhoff, et al., 2016 | To characterize the psychological factors associated with persistent symptoms after pediatric concussion | 179, 13.4 | Longitudinal cohort study | - Score distributions were significantly worse on the STAIC among children  with delayed symptom resolution compared with children with early symptom resolution. |
| 3 | Rieger et al., 2019 | To compare adolescent students with and without concussion on a battery of academic, neurocognitive,  and socioemotional measures and assess the aftereffects of concussion across domains of functioning | 48, 14.89 | Case-control | - There are significant findings on self-report measures for socioemotional perceptions, with concussion group having significantly higher T scores on internalizing symptoms composite (atypicality, locus of control, social stress, anxiety, depression) and emotional symptoms index (anxiety, social stress, depression, sense of inadequacy, self-reliance, and self-esteem subscales) than controls |
| 4 | Schilling et al., 2020 | To examine differences in symptom burden and profile scores among concussed children aged 10-18 with a prolonged recovery as compared to their counterparts with a typical recovery | Total: 4380  10–12: 584  13–15: 1927  16–18: 1869 | Retrospective case-control study | - The prolonged recovery group had significantly higher scores on all five symptom profiles, including anxiety (p < 0.001), than the typical recovery group. Older age was a major factor in the anxiety group. |
| 5 | Stazyk et al., 2017 | To explore the risk and possible predictors of developing significant depressive symptoms in children recovering from  concussion. | Depressed: 20, 14.59  Not Depressed: 72, 14.71 | Prospective cohort study | - In comparing children depressed vs. those not depressed, there were no significant differences in reports of multiple head injuries, cause of injury, or hospital admissions. - Significant differences in number of concussion symptoms as reported on PCSI between depressed and not depressed. - Children with depression had higher initial clinical PCSI mean scores and higher PCSI scores reported on third clinical visit - Significant correlation between depression scores and the number of concussion symptoms reported on the PCSI-Time I and Time 3 indicating a linear relationship. - Five independent variables (gender, number concussions, admission to hospital, PCSI Time 1 score, and experiencing symptoms over 3 months was significant to depression, thus the odds for children to have a positive depression score at a mean of 6 months after concussion was associated with those 5 variables. - Strongest predictor of the model was high PCSI scores and  admission to hospital - High depression score increased by a factor of 11 if the child was admitted  to hospital compared to those not admitted - Early PCSI is 1.32, meaning that every one-point increase in symptom scores, the odds of being depressed increases by a factor of 1.32 |
| 6 | Stein et al. (2017) | To investigate the symptom trajectories of depressive and post-concussive symptoms  in slow-to-recover adolescents to  understand how the two sets of  symptoms are related. | 49, 15 | Randomized clinical trial of collaborative care intervention | - PCS and depressive symptoms improve over time - Declines in depressive symptoms were greatest between the second and third (3 months after enrolment) measurement occasions. Initial depressive symptoms were not related to change in post-concussive symptoms over time - Initial level of depressive symptoms was 9.66 and growth of depressive  symptoms decreased on average by -5.92. - Participants with a higher depressive symptom level at time of enrolment were associated with a sharper decline over time - Participants with higher PCS tended to have higher levels of depressive symptoms - Decreases in PCS were strongly related to decreases in depressive symptoms - Participants with higher initial levels of depressive symptoms tended to decline more in depressive symptoms over time - Results indicate a significant effect of initial PCS status predicting change in depressive symptoms over time (higher PCS, greater the predicted decrease in depressive symptoms over time). |

**Table 7: Detailed Results Chart (Adult)**

| **Adult** | | | | | |
| --- | --- | --- | --- | --- | --- |
| Count | Author, year | Purpose | Population (n, mean age) | Design | Key findings |
| 1 | Andre-Morin et al., 2017 | To explore female university athletes' experiences with protracted concussion symptoms, including their perceptions of the factors that impeded or facilitated  their recovery | 5, university age | Interpretive  Phenomenological  Analysis | - Heightened emotions such as frustration, mood swings, depression, and changes in behaviour. Suicidal thoughts. |
| 2 | Agtarap et al., 2019 | To evaluate the structure of post-concussive and psychological symptoms using data from Army Combat Teams, to investigate the symptom domain of PPCS | 1229, 24.99 | Retrospective longitudinal study | - Soldiers with high deployment stress exposure had significantly greater PCS, Posttraumatic Stress, and Depression factor scores. - Depression more prevalent in mTBI than very mild TBI but not significant. |
| 3 | Ahman et al., 2013 | To characterize the long-term consequences of mTBI regarding post-concussion symptoms, PTSD, and QoL  To investigate differences between men and women | 163, 30.8 | Retrospective mixed  methods study | - One of the most common symptoms was depression (47.2%) - Women had a higher prevalence of headaches (60.3%) and depression (47.2%) in comparison with men (44.2%, 47.2%, p = 0.043 and p = 0.029, respectively). - 65.4% had intrusion symptoms and 59.5% had avoidance symptoms. - Moderate to severe stress reported in 10% of men and 14% of women. - Women reported significantly higher depression scores and stress scores. - Mental limitations stemming from the injury (irritable, anger). |
| 4 | Donnell et al., 2012 | To examine the incidence of satisfying only the symptom-based criteria for PCS in a population-based sample of  community-dwelling Vietnam-era US military veterans | 4662, 37.8 | Retrospective  cross-sectional study | - 50% of mTBI veterans were diagnosed with GAD. - 57% of mTBI veterans were diagnosed with MDD. - 75% of comorbid GAD and MDD met criteria for PCS v. 80% with comorbid mTBI. |
| 5 | Eman Abdulle et al., 2020 | To investigate the role of postconcussive symptoms, mood, post-traumatic stress, and coping (assessed at 2 weeks postinjury) on functional outcomes measured 6 months after sustaining an mTBI in elderly patients | 162, 71 | Longitudinal  observational cohort  study | - Higher prevalence of depression was found in patients with an incomplete recovery (20%) as compared to patients with complete recovery (7%) - Higher depression scores (OR = 0.87, p = 0.005) and higher HISC scores (OR = 0.91, p = 0.03) were significantly associated with decreased odds of complete recovery. |
| 6 | Greenburg et al., 2020 | To test the strength and reliability of cognitive (pain catastrophizing) and behavioural (limiting behaviours)pathways mediating the relationship between anxiety and postconcussion  symptoms among patients with mTBI | 57, 47.08 | Cross-sectional  observational study | - Total effect of anxiety of PCS were significant, suggesting an direct effect of anxiety on PCS through pain catastrophizing - The total effect of anxiety on PCS were significant, suggesting a direct effect of anxiety on PCS through limiting behaviour. - Anxiety significantly associated with both limiting behaviour and pain catastrophizing through direct and indirect paths, indicating multiple mediation effects were partial - Anxiety strongly correlated with postconcussion symptoms, in line with both simple and multiple mediation of pain catastrophizing and behaviour limiting paths. |
| 7 | Lange et al., 2011 | To examine the influence of depression on postconcussion symptom reporting in patients following mTBI | 190, 37.8 | Cross-sectional observational  study | - Minimal significant differences in PCSS between mTBI with depression and depression outpatients - Used to classify participants into categories not for overall results - Patients who experience MTBIs and who have a postinjury recovery course complicated by significant depression report more postconcussion symptoms, and more severe symptoms, than (a) outpatients with depression, and (b) patients with MTBIs who do not have significant symptoms of depression |
| 8 | Maruta et al., 2016 | To characterize cognitive deficits of adult patients who had persistent symptoms after a concussion and determine whether the original injury retains associations with these deficits after accounting for  the developed symptoms that overlap with PTSD and depression | 173, 34.9 | Longitudinal cohort study | - Patient CES-D average score (mean, sd) = 19.8, 13.6 - Patient group endorsed significantly higher level of depression than controls - Some correlations of cognitive performance metrics to  depression symptoms reached statistical significance, although, they were generally moderate or weak. |
| 9 | Mooney & Speed, 2001 | To further increase one's understanding of the factors that may be responsible for  failure to recover after mTBI  To investigate the relationship between psychiatric comorbid conditions and outcome after mTBI | 80, 31.1 | Prospective, descriptive  analysis | - Depression after injury = 35 (44%) - Anxiety after injury = 19 (24%) - Total psychiatric conditions = 60 (76%) - New conditions that developed post injury, and had a two-threefold increase in the prevalence of psychiatric conditions after injury, most increase took form of anxiety and depression - Most of the participants with no psychiatric condition made a good recovery, suggesting that recovery may be substantially driven by whether or not psychiatric comorbidity is present. |
| 10 | Mooney et al., 2005 | To study the recovery variables that relate to outcome after mTBI | 67, 41.36, | Prospective descriptive analysis | - 56 subjects (85%) had a psychiatric diagnosis, which is a considerable increase over 54% preinjury lifetime psychiatric diagnoses. - BDI and PCS symptoms were significantly correlated Specifically, relationship of depression to PCS remained significant while controlling for pain. |
| 11 | Morissette, et al., 2011 | To examine the relations among TBI, persistent PCS, and symptoms of PTSD and depression among returning  OEF/OIF veterans | 213, 38 | Longitudinal cohort study | - Veterans with positive TBI had more symptoms of depression than negative TBI veterans. |
| 12 | Oldenburg et al., 2018 | To investigate aspects of emotional reserve,  psychological variables and psychiatric vulnerability and  their association with a restrictively defined PCS group 1-year postinjury in the same study group | 94, 36.7 for recovered group | Prospective cohort study | - Patients who developed PCS reported higher levels of anxiety than the recovered group, and also experienced higher levels of depression than the recovered group. |
| 13 | Ponsford et al., 2019 | To investigate the nature of persistent symptoms, work/study outcomes, anxiety and quality of life and factors associated with persistent symptoms following injury,  including the impact of receiving information about mild traumatic brain injuries in the emergency department | 343, 54.21 | Clustered randomized trial | - 14.9% of participants scored >7 on HADS anxiety scale, indicating clinically significant anxiety symptoms at follow-up, where 7.2% scored as mild, 5.6% as moderate, and 2.1% as severe - Anxiety was statistically significantly correlated with PCS after adjusting for cluster, whereby higher anxiety was  associated with greater reporting of persistent novel post-injury PCS at follow up. |
| 14 | Rapoport et al., 2003 | To assess the association of major depression with behavioural outcome following mild traumatic brain injury | 170, 41.3 | Case-control | - Participants with major depression reported higher levels of psychosocial dysfunction on Rivermead Head Injury Follow Up Questionnaire and greater symptoms of postconcussive disorder on Rivermead Post Concussion Symptoms Questionnaire - Relationships were significant even after mechanism of injury was controlled - Subjects with depression reported higher levels of PCS even excluding symptoms that overlap with major depression. |
| 15 | Theadom et al., 2016 | To determine whether people sustain any persistent effects 1 year after mTBI, and to identify the predictors of health outcomes | 341, 37.5 | Community- based, longitudinal population study | - Levels of anxiety and depression were comparable with the general population. |
| 16 | Yang et al., 2007 | To examine PPCS during the disease course and to  determine whether PPCS adversely affect outcome | mTBI 115, 36.7  Control 40, 34.85 | Prospective,  cohort and  controlled study | - The psychosocial symptoms, such as depression and irritability, were significant at the late after injury stage (4 and 8 weeks). - The analysis showed that headache (4.00, p 0.05), dizziness (4.00, p 0.05), fatigue (4.92, p 0.05), poor vision (75.98, p 0.001), nausea (37.66, p 0.001), and depression (37.66, p 0.001) were significantly associated with GOSE score. |
| 17 | Waljas et al. (2015) | To examine multiple biopsychosocial factors relating to post-concussion symptom (PCS) reporting in patients  with mild traumatic brain injuries, including structural and microstructural neuroimaging. | mTBI: 126, 37.8  Control:  36, 36.9 | Prospective, longitudinal inception cohort design | - Correlation between BDI-II subscales and RPSQ total scores in the control group was 0.48. - At one month, the mTBI group had higher mean BDI-II subscale scores than control group, but not different enough to be called significant after considering multiple comparisons. - At one year, the mTBI group's BDI-II subscales scores did not  differ from the control group mean subscale BDI-II score. - Patients who met ICD-10 criteria for PCSS had significantly higher BDI-II subscale total scores than patients who did not meet ICD-10 criteria at one month based on “mild or greater” symptom reporting and “moderate or greater” symptom reporting. |
| 18 | Lange et al., 2015 | To examine the relation between microstructural architecture of white matter, as measured by DTI, and postconcussion symptom reporting 6-8 weeks following mTBI. | No PCS: 52, 34.1 PCS: 20, 34.1 | Prospective cohort | - PPCS group had higher total scores on BDI-II and BAI compared with no PPCS - Only two of six variables were significant predictors of group membership (depression and BAI) - 83.3% of participants were correctly classified using the depression and BAI total scores alone. - Classification scores were substantially higher when predicting no PPCS group compared with PPCS group. |
| 19 | Doroszkiewicz et al., 2021 | To evaluate the prevalence of clinical symptoms of anxiety and depression and the relationship between PCS and quality of life in patients examined at the Canadian  Concussion Center | 105, 33.46 | Prospective,  longitudinal population-based study | - 37 (35.2%) of patients had clinically elevated symptoms of depression, anxiety, or both (clinically elevated beyond the normal range) - 8 (7.6%) had symptoms of depression alone - 8 (7.6%) had symptoms of anxiety alone - 21 (20%) had symptoms of both depression and anxiety - The majority of patients in this population did not endorse  elevated symptoms of depression and/or anxiety - Increased number of previous concussions correlated to median depression, anxiety, and stress scores - No association between mechanism of injury or sex - Patients with elevated depression, anxiety, or both exhibited poorer mean QoL outcomes (WHOQOL-BREF), indicating that depression and anxiety in PCS can endure for years and are associated with diminished QoL |
